# Interaction Between Root Exudates of the Poisonous Plant *Stellera chamaejasme* L. and Arbuscular Mycorrhizal Fungi on the Growth of *Leymus Chinensis* (Trin.) Tzvel

**DOI:** 10.3390/microorganisms8030364

**Published:** 2020-03-04

**Authors:** Xinrui Zhu, Xiaote Li, Fu Xing, Chen Chen, Guohui Huang, Ying Gao

**Affiliations:** 1 Institute of Grassland Science, Northeast Normal University, Changchun 130024, China; zhuxr237@nenu.edu.cn (X.Z.); lixt900@nenu.edu.cn (X.L.); chenc049@nenu.edu.cn (C.C.); huanggh699@nenu.edu.cn (G.H.); 2 Key Laboratory of Vegetation Ecology, Ministry of Education, Changchun 130024, China

**Keywords:** root exudates, allelopathy, AMF, interspecific relationship, degraded grasslands

## Abstract

The growth of a large number of poisonous plants is an indicator of grassland degradation. Releasing allelochemicals through root exudates is one of the strategies with which poisonous plants affect neighboring plants in nature. Arbuscular mycorrhizal fungi (AMF) can form a mutualistic symbiosis with most of the higher plants. However, the manner of interaction between root exudates of poisonous plants and AMF on neighboring herbage in grasslands remains poorly understood. *Stellera chamaejasme* L., a common poisonous plant with approved allelopathy, is widely distributed with the dominant grass of *Leymus chinensis* in the degradeds of Northern China. In this study, we investigated the addition of *S. chamaejasme* root exudates (SRE), the inoculation of AMF, and their interaction on the growth and tissue nitrogen contents of *L. chinensis*, the characteristics of rhizosphere AMF, and soil physicochemical properties. Results showed that SRE had significant effects on ramet number, aboveground biomass, and total nitrogen of *L. chinensis* in a concentration dependent manner. Additionally, SRE had a significant negative effect on the rate of mycorrhiza infection and spore density of the AMF. Meanwhile, the addition of SRE significantly affected soil pH, electrical conductivity, available nitrogen (AN), available phosphorus (AP), total nitrogen (TN), and total carbon (TC) contents; while neither inoculation of AMF itself nor the interaction of AMF with SRE significantly affected the growth of *L. chinensis*. The interaction between AMF and SRE dramatically changed the pH, AP, and TC of rhizosphere soil. Therefore, we suggested SRE of *S. chamaejasme* affected the growth of *L. chinensis* by altering soil pH and nutrient availability. AMF could change the effect of SRE on soil nutrients and have the potential to regulate the allelopathic effects of *S. chamaejasme* and the interspecific interaction between the two plant species. We have provided new evidence for the allelopathic mechanism of *S. chamaejasme* and the regulation effects of AMF on the interspecific relationship between poisonous plants and neighboring plants. Our findings reveal the complex interplay between the root exudates of poisonous plants and rhizosphere AMF in regulating population growth and dynamics of neighboring plants in degraded grassland ecosystems.

## 1. Introduction

Grassland occupies approximately one third of the land area of China, and provides great ecological and economic value to the country [1,2]. However, grassland degradation has exacerbated during the past 50 years due to intense human disturbance, especially overgrazing [3]. One important indicator of grassland degradation is increasing abundance of poisonous plants. These plants commonly accumulate and exude secondary metabolites that are toxic to livestock or humans [4,5,6]. Moreover, poisonous plants directly compete with palatable forage plants for resources such as water and mineral nutrition [7,8].

*Stellera chamaejasme* L. is a common poisonous plant species in the degraded grasslands of northern China [9], which even becomes the dominant species in some extremely degraded grasslands [10,11]. A growing number of studies have shown that *S. chamaejasme* can exert allelopathic substance, and inhibit the germination and seedling growth of other plants [12,13,14]. The fresh roots, stems, and leaves of *S. chamaejasme*, as well as the comminuted substance from different plant parts, have been found to have negative effects on many important forage plant species, including *Medicago sativa* L., *Astragalus adsurgens* Pall., *Elymus dahuricus* Turcz., and *Elymus sibiricus* L. [12,13]. The root has stronger allelopathic influence than the stem and leaf. For example, the water and alcohol extracts of *S. chamaejasme* roots can inhibit germination of *Arthraxon hispidus* (Thunb.) Makino by 5%–15% and seedling growth of *Galium verum* L. by more than 50% [14]. In parallel, many secondary metabolites such as coumarins, flavonoids, lignans, and terpenoids have been isolated and identified from *S. chamaejasme* [15,16,17,18]. Mechanistically, allelochemicals could affect photosynthesis, respiration, and the metabolism of certain groups of proteins and nucleic acid of other plants, which in turn regulate their growth [17,19]. Some studies have shown that allelopathic substance can alter soil pH and nutrient availability, e.g., terpenoids may play an important role in the inhibition of nitrification [20]. In *S. chamaejasme*, the flavonoids secreted by the root could indirectly increase the edaphic available phosphorus (AP) [21]. However, despite these significant advances, little is known about whether and how allelopathic substance of *S. chamaejasme* affects soil characteristics and the growth of neighboring grasses. 

The interspecific competition is a classical topic in ecology [22]. Previous studies on interspecific competition in plants have usually focused on competition for nutrients [23,24], or the niche overlap [25]. In addition to poisonous plants, the infection of arbuscular mycorrhizal fungi (AMF) is another important biotic factor in structuring plant communities and regulating the interspecific relationship [26]. A growing body of studies has shown that AMF exist widely in terrestrial ecosystems and form symbionts with plants to promote nutrients uptake and increase their utilization rate [27,28,29,30]. AMF could mediate the interaction between plant species and significantly affect the distribution of individual size in plant population and plant community structure [31,32,33,34,35]. Therefore, AMF may play an essential role in regulating the relationship between plants, including intra- and inter-specific competitions [36,37]. However, whether AMF also play such a role in regulating the interactive relationships between *S. chamaejasme* and neighboring plants has been rarely investigated.

*Leymus chinensis* L. is a rhizomatous perennial grass, with a high AMF infection rate on its root system [38]. This species is a primary forage grass for livestock and often coexists with *S. chamaejasme* in the degraded grasslands of northern China [39]. With grassland degradation, *S. chamaejasme* can become a dominant species in the community and compete with neighboring plants for space and resources [7,8]. However, the interspecific relationship between this poisonous plant and *L. chinensis* and the mechanisms underlying interactions are remain poorly understood. Interestingly, a field study carried out in the grasslands of Inner Mongolia, China, found that there was no AMF infection in the root of *S. chamaejasme* [40]. Studies have found that the rhizosphere soils of *S. chamaejasme* had lower relative abundance of AMF [41]. These findings indicated that the root exudates of *S. chamaejasme* may be able to inhibit AMF in the rhizosphere. However, little is known about whether poisonous plants can interact with AMF infection to regulate the growth of neighboring plants.

In this study, we performed a two-factor interactive experiment to test how root exudates of *S. chamaejasme* and AMF inoculation, as well as their interaction, could affect the growth of *L. chinensis*, the characteristics of rhizosphere AMF, and soil physicochemical properties. We hypothesized that (i) the root exudates of *S. chamaejasme* would have negatively allelopathic effects on the growth of *L. chinensis* by altering soil physicochemical properties; (ii) there would be an interactive effect of AMF and root exudates on the growth of *L. chinensis;* and (iii) AMF could mediate the allelopathic effects of *S. chamaejasme* on *L. chinensis* via changing soil pH and nutrient availability. 

## 2. Material and Methods

### 2.1. Material Preparation 

*L. chinensis* is naturally highly colonized by *Glomus* spp. of AMF in the fields [38,42], so *Glomus tortuosum* Schenck and Smith were selected as the AMF inoculation agent in our study. The stain was purchased from the Germplasm Repository of AMF of Beijing Academy of Agricultural and Forestry Sciences. *M. sativa* L. was used for fungus propagation. The expanding propagation of AM fungal spores was completed from June to August 2016 in a greenhouse, which was located in the College of Life Sciences, Northeast Normal University, Changchun City, China (43°51′38″ N, 125°19′26″ E). 

The seeds of *L. chinensis* were collected from natural grasslands in Changling County, Jilin Province in the fall of 2016. After disinfection with 0.5% potassium permanganate solution, *L. chinensis* seeds were sown in the nursery with sterilized soil and germinated in a greenhouse (illuminated for 12 h, temperature is 26 °C in the day and 22 °C in the night) in early May 2017. After the seedlings grew to the trefoil stage, they were transplanted into the pots containing sterilized soil and sand (volume ratio of soil and sand, 4:1). Three plant individuals were planted in each pot. The soil was collected from the native grasslands of *L. chinensis.* The soil and sand were sterilized at 121 °C for 30 min before combining together. The weight of soil substrates in each pot was identical, and the pot size was 18 cm in diameter and 20 cm in height. 

*S. Chamaejasme,* a poisonous perennial plant, widely distributes in the overgrazed steppe of Inner Mongolia of China [9]. Root exudates from *S. chamaejasme* were collected by in vivo culture as described previously [43,44]. Briefly, entire living *S. chamaejasme* plants were taken without hurting their roots from the degraded grassland which is located in Tianshan town, Chifeng city, eastern Inner Mongolia. The roots were washed by water to remove debris like soil and gravel, followed by sterilization by 70% alcohol. We used 20 *S. chamaejasme* plants aged eight-year-old for collecting root exduates. Each plant was placed in a special tin barrel containing 6 L Hoagland’s nutrient solution, with stems and leaves exposed to normal light. Seven days were allowed for the secretion of root exudates into the culturing solution and then mixed all of the culturing solution to be recovered and condensed by a reverse osmosis membrane (Model: TW30-1812-50, Dow, IA, USA) suction filter. The roots of all *S. chamaejasm* were dried at 105 °C for 48 h to measure the root biomass. Finally, the culturing solution was condensed to a specified level of 0.1 g/mL (i.e.,0.1 g is the root biomass of *S. chamaejasme*). The prepared root exudates were stored in a refrigerator at 4 °C.

### 2.2. Experimental Design and Treatments

The experimental design was a completely randomized two-factor interaction. AMF treatment included two levels: inoculation with and without AMF. The treatment of *S. chamaejasme* root exudates (SRE) had three concentration levels: the original solution (SRE_0_, mass concentration 0.1 g/mL), five-fold dilution (SRE_5_, mass concentration 0.02 g/mL), and twenty-fold dilution (SRE_20_, mass concentration 0.005 g/mL). The Hoagland’s nutrient solution was used as a control (CK). The dilutions (SRE_5_ and SRE_20_) were obtained by diluting the original solution with the Hoagland’s nutrient solution. These concentrations of root exudates (SRE_0_, SRE_5_ and SRE_20_) were designed basing on the methods and results of the previous studies on *S. chamaejasme* [45,46]. Combining the two factors, there were eight treatments in total (including CK), and each treatment had eight replicates (pots). The addition of root exudates and the inoculation with the AMF were carried out in July, 2017. The seedlings of *L. chinensis* had grown for two months after seed germination, whose height was about 12 cm. The spores collected by a 100 g expansion matrix of *G. tortuosum* were suspended to 100 mL in distilled water, and then were poured into all the pots simultaneously. For the negative control, the same amount of distilled water was poured into the pots without the AMF inoculation. There were approximately 400 AM fungal spores in each pot. The root exudate with the above three concentration gradients was added with 100 mL per pot, and the same amount of nutrient solution was poured in CK. The addition of root exudate treatment was performed every ten days for a total of four times.

Moreover, to mimic the natural composition of the microbial communities, all the pots were treated with 120 mL filtrate from non-sterile grassland soils. The filtrate was obtained by passing a 50:60 suspension of the soil inoculum through a filter paper (the pore size < 10 μm) to remove AM propagules [47]. The pots were placed in the greenhouse and arranged randomly. During the experiment, an equal amount of distilled water (100 mL) was added to each pot twice a week to keep soil moisturized.

### 2.3. Measurements of the Characteristics of Plant, AMF, and Soil

Ramet number, total rhizome length, above-ground and underground biomass of *L. chinensis* were measured on 19 August 2017. More specifically, ramet number of *L. chinensis* in each pot was recorded, and then the aboveground and underground parts were separated. Subsequentially, the underground part was washed with water, and then total rhizome length was measured as described previously [48]. The aboveground and underground parts were dried at 105 °C for 15 min (keeping the chemical composition of plant samples unchanged), then at 65 ºC for 72 h for measuring biomass, respectively. The dried plant samples were ground using a grinding miller to make the particles less than 1 mm. Total aboveground nitrogen (shoot TN) and total underground nitrogen (root TN) of *L. chinensis* were measured on a Kjeldahl apparatus (Kjeltec 8400, FOSS, Hillerød, Denmark) after digesting the plant samples with sulphuric acid (ω ≈ 98%). 

The rhizosphere soil of *L. chinensis* was collected by the buffeting method to detect the AM fungal spore density and soil chemical properties. A small amount of fresh fibrous roots were selected and stored in a 4-degree refrigerator for detecting infection rate of AMF in the root systems of *L. chinensis*. Finally, the biomass of the fibrous roots was incorporated into underground biomass. Mycorrhizal infection was determined by a mycorrhizal staining method [49,50]. Spore density of AMF in the rhizosphere soil was determined by an improved wet sieve-sucrose gradient centrifugation method [51].

A portion of the soil samples was air-dried, ground and then sieved through a 0.15 mm mesh to measure soil pH, electrical conductivity (EC), the contents of available nitrogen (AN), total nitrogen (TN), available phosphorus (AP), total phosphorus (TP), and total carbon (TC). Soil pH and EC were measured in water suspension (water/soil = 2.5:1) using a pH meter (pH S-3C, INESA, Shanghai, China). EC was determined using an electrical conductivity instrument (DDS-307, INESA, Shanghai, China). AN was measured using a flow analyzer (Futura, AMS, Frépillon, France), and the pretreatment was completed using the alkaline hydrolysis and diffusion extraction method [52]. TN was measured on the Kjeldahl apparatus (Kjeltec 8400, FOSS, Hillerød, Denmark). AP was extracted with 0.5 mol·L^1−^ NaHCO_3_ solution (pH 8.5) and was determined using Molybdenum antimony and the scandium colorimetry method on a spectrophotometer (UV5−500, METASH, Shanghai, China) [52]. TP was measured using an automatic discontinuous chemical analyzer (Smartchem 450, AMS, Rome, Italy) after digesting the soil samples with sulfuric acid-perchloric acid. TC was determined using the total organic carbon analyzer (Vario TOC, Elementar, Langenselbold, Germany).

### 2.4. Statistical Analysis

Before the ANOVA was carried out, all the data were tested for variance homogeneity (Levence-test) and normal distribution (Kolmogorov–Smirnov test). A two-way analysis of variance (ANOVA) (95% CI) was used to determine the differences of the interactive effects of the two factors (i.e., the addition of root exudates and inoculation with and without AMF on the characteristics of *L. chinensis* and soil properties). One-way ANOVA was also used to determine the significant effect of different concentration levels of *S. chamaejasme* root exudates. T-test was used to determine the significant difference in the growth characteristics of *L. chinensis*, shoot TN, root TN, and soil characteristics between with and without AMF inoculation, respectively. The multivariable stepwise regression analysis was used to identify the factor that could effectively explain the correlation among the characteristics of soils, AMF and *L. chinensis*. The statistical analysis was performed using SPSS software (SPSS Statistics 20.0, Chicago, IL, USA). All figures were produced using SigmaPlot software (Sigmaplot 12.5, Systat Software Inc., San Jose, CA, USA).

## 3. Results

### 3.1. The Growth of L. chinensis 

#### 3.1.1. Ramet Number and Rhizome Length

**Figure 1:**
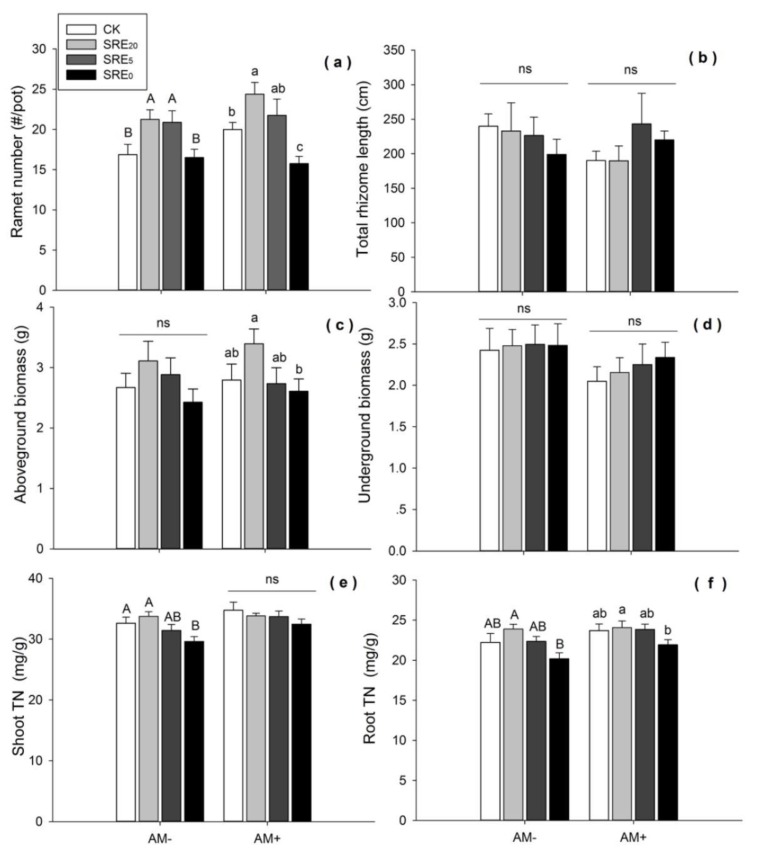
Effects of the addition of *S. chamaejasme* root exudate (SRE) and inoculation without (AM−) and with (AM+) arbuscular mycorrhizal fungi (AMF) on (**a**) ramet number, (**b**) total rhizome length, (**c**) aboveground biomass, (**d**) underground biomass, (**e**) the content of shoot total nitrogen (TN), (**f**) the contene of root TN of *L. chinensis.* Values were means ± SE from eight repeat samples. Different capital letters, or lowercase letters indicate significant differences (*p* < 0.05, LSD) among the three SRE concentrations and CK within AM− or AM+ treatment, respectively. ns = no significance. SRE_0_, SRE_5_, and SRE_20_ represented the original solution, five-fold dilution, and twenty-fold dilution of *S. chamaejasme* root exudates. The mass concentration within these three SRE treatments was 0.1 g/mL, 0.02 g/mL, and 0.005 g/mL, respectively.

Within no AMF inoculation (AM−), SRE_20_ and SRE_5_ significantly increased ramet number of *L. chinensis* (*p <* 0.05), while there was no significant difference between SRE_0_ and the CK. With AMF inoculation (AM+), SRE_20_ significantly increased, and SRE_0_ significantly decreased ramet number (*p <* 0.05) (Figure 1a). Therefore, the treatments with lower root exudate concentrations showed positive effects on ramet number, whether *L. chinensis was* inoculated with AMF or not. However, high concentration of root exudates had an inhibitory effect on ramet number under inoculation of AMF.

There was no significant difference in total rhizome length of *L. chinensis* among all concentrations of SRE within the treatment groups of AM− and AM+, respectively (Figure 1b).

#### 3.1.2. Aboveground and Underground Biomass

SRE_20_ tended to increase the biomass of *L. chinensis* within non-inoculated AM−, but there was no significant difference among the three levels and CK. When AMF was inoculated, aboveground biomass of *L. chinensis* decreased gradually with the increasing levels of root exudates, and SRE_0_ significantly reduced aboveground biomass compared with SRE_20_ (*p <* 0.05) (Figure 1c). However, SRE_0_ did not significantly reduce this growth trait compared with CK. There were no significant differences in the underground biomass of *L. chinensis* between SRE and AMF treatments (Figure 1d). It seemed that underground biomass was insensitive to the addition of SRE and AMF inoculation.

Moreover, the results of the two-way ANOVA showed that SRE had significant effects on ramet number (*p <* 0.001) and aboveground biomass (*p <* 0.05) of *L. chinensis*, while AMF inoculation and their interaction of SRE × AMF had no significant effects on any growth parameters of *L. chinensis* (Table 1).

### 3.2. Total Nitrogen Content of Shoots and Roots

When AMF were not inoculated, SRE_0_ significantly reduced shoot TN of *L. chinensis* (*p <* 0.05). When AMF were inoculated, there was no difference in shoot TN among the three levels of root exudates and CK (Figure 1e). In general, higher concentration of root exudates had a negative effect on shoot TN in *L. chinensis*.

SRE_0_ significantly reduced root TN compared with the SRE_20_, while there were no significant differences between the three concentrations of root exudates and CK (Figure 1f). With or without AMF inoculation, there was no significant difference in root TN between all concentrations of root exudates with CK. In addition, T-test results showed that AMF inoculation significantly increased shoot TN (*df* = 62, *p* = 0.01) and root TN (*df* = 62, *p* = 0.042) compared with the condition without AMF inoculation.

The variance analysis showed the significant effects of SRE on shoot TN (*p <* 0.05) and root TN (*p <* 0.01) (Table 1). However, AMF treatment had a significant effect only on shoot TN (*p <* 0.01). Shoot TN of *L*. *chinensis* was significantly higher in the condition of AM+ than AM− at the concentration of SRE_0_ (*df* = 14, *p* = 0.029). This indicated that AMF inoculation had a positive effect on the nitrogen accumulation in the shoots of *L. chinensis*.

### 3.3. AM Fungal Characteristics

#### 3.3.1. Infection Rate

**Figure 2:**
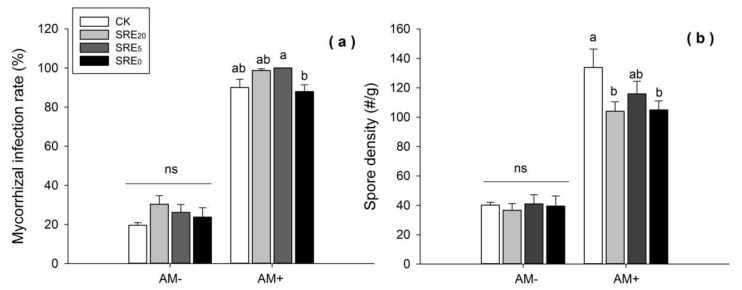
Effects of the addition of *S. chamaejasme* root exudate (SRE) and inoculation without (AM−) or with (AM+) AMF on the (**a**) mycorrhizal infection rate and (**b**) spore density of AMF. Values were means ± *SE* from eight repeat samples. Different lowercase letters above bars indicate significant differences (*p* < 0.05, LSD) among the treatments within AM− or AM+ treatment. ns = no significance. SRE_0_, SRE_5_, and SRE_20_ represented the original solution, five-fold dilution, and twenty-fold dilution of *S. chamaejasme* root exudates. The mass concentration within these three SRE treatments was 0.1 g/mL, 0.02 g/mL, and 0.005 g/mL, respectively.

The root exudate treatment had no significant effects on the infection rate in the non-inoculated (AM−) *L. chinensis* root system. SRE_20_ and SRE_5_ tended to increase infection rate compared with the CK treatment under the condition of *G. tortuosum* inoculation (AM+), while the increase was not significant at *p <* 0.05 statistical level (Figure 2a).

#### 3.3.2. Spore Density

Under the AM− condition, spore density was not significantly affected by different concentration levels of root exudates (Figure 2b). However, spore density within SRE_0_ and SRE_20_ was significantly lower than that of CK under AM+ (*p <* 0.05). T-test results showed that AM+ significantly increased the mycorrhizal infection rate of *L. chinensis* (*df* = 62, *p* = 0.000) and AM spore density (*df* = 34, *p* = 0.000) compared with AM−.

The analysis of variance showed that SRE had a significant effect on the infection rate and spore density of AMF (*p* < 0.01). Similarly, AMF also had a significant effect on the above two parameters (*p <* 0.001). Yet, no obvious influence of their interaction between the two treatments (SRE × AMF) on the parameters of AMF was found (Table 1).

### 3.4. Soil Characteristics

**Figure 3:**
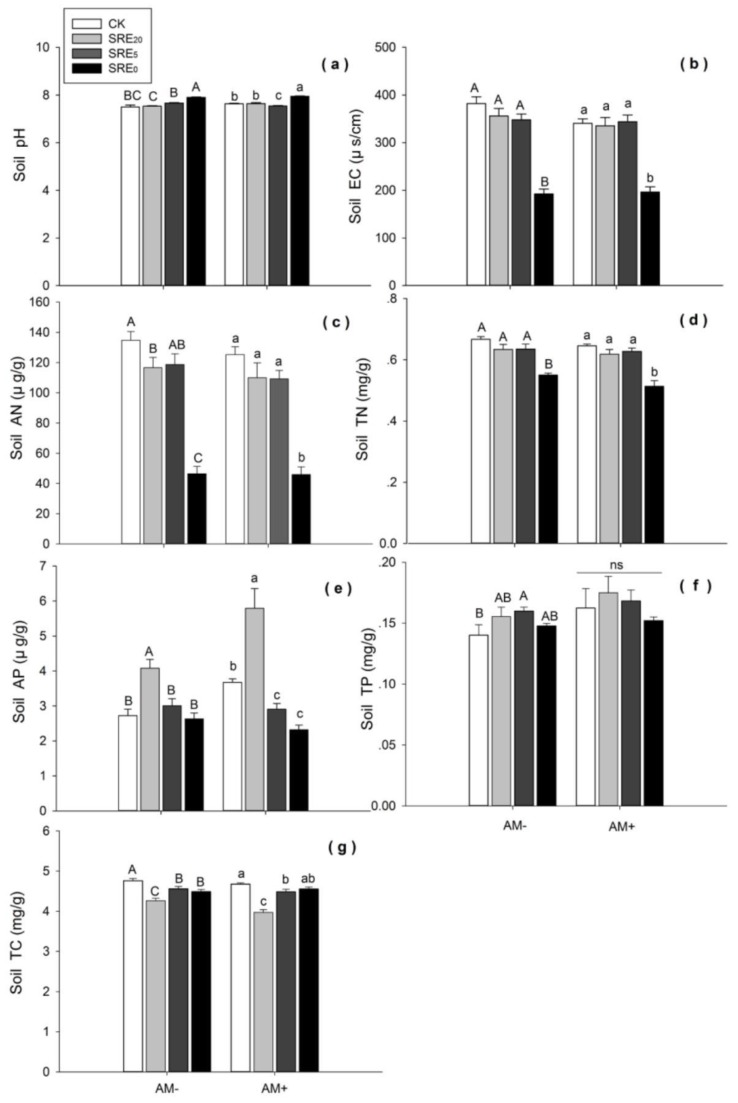
Effects of the addition of *S. chamaejasme* root exudate (SRE) and inoculation without (AM−) or with (AM+) AMF on (**a**) soil pH, (**b**) soil electrical conductivity (EC), (**c**) soil available nitrogen (AN), (**d**) soil total nitrogen (TN), (**e**) soil available phosphorus (AP), (**f**) soil total phosphorus (TP), (**g**) soil total carbon (TC). Values were means ± SE from eight repeat samples. Different capital letters, or lowercase letters indicate significant differences (*p* < 0.05, LSD) among the three SRE concentrations and CK within AM− or AM+ treatment, respectively. SRE_0_, SRE_5_, and SRE_20_ represented the original solution, five-fold dilution, and twenty-fold dilution of *S. chamaejasme* root exudates. The mass concentration within these three SRE treatments was 0.1 g/mL, 0.02 g/mL, and 0.005 g/mL, respectively.

SRE_0_ significantly increased soil pH compared with CK (*p <* 0.05) under the condition of AM−, while no significant effects of SRE_20_, and SRE_5_ on the pH were found. Under the AMF inoculation condition, SRE_0_ significantly increased but SRE_5_ decreased soil pH, respectively (*p <* 0.05) (Figure 3a).

Whether *L. chinensis* was inoculated by AMF or not, the high concentration of SRE_0_ significantly decreased soil EC (*p <* 0.05), AN (*p <* 0.01), and TN (*p <* 0.05) (Figure 3b–d). However, there was no significant difference in EC and TN among SRE_20_, SRE_5_ and CK levels (Figure 3b,d).

SRE_20_ showed a remarkable increasing effect on soil AP compared with CK (*p <* 0.05) in both AM− and AM+ groups (Figure 3e). However, SRE_5_ and SRE_0_ significantly decreased soil AP compared with CK under the condition of AMF inoculation (*p <* 0.05), respectively. Lower concentrations of root exudates (SRE_20_) had a significantly positive effect on soil AP, while only negative effects of higher concentration were found in AM+ group.

Compared with CK, SRE_5_ significantly increased soil TP (*p <* 0.05) in AM− group, while SRE_0_ and SRE_20_ had no significant effects (Figure 3f). Under the condition of AMF inoculation, there was no significant difference among the three SRE concentrations and CK (*p <* 0.05). In addition, T-test results of AM+ and AM− groups showed that AMF inoculation significantly increased soil TP (*df* = 62, *p* = 0.040).

SRE_20_, SRE_5_, and SRE_0_ treatments significantly decreased soil TC compare with CK (*p <* 0.05) when *L. chinensis* was not inoculated with AMF. SRE_20_ and SRE_5_ significantly decreased soil TC compared in AM+ group (*p <* 0.05), while there was no significant difference between SRE_0_ and CK (Figure 3g). These results showed SRE_20_ had a stronger inhibitory effect on soil TC than the other two levels of SRE.

According to the two-way ANOVA, there were significant effects of SRE on soil pH, EC, AN, TN, AP and TC (*p <* 0.001). Additionally, AMF inoculation had significant effects on soil TN (*p <* 0.05), AP (*p <* 0.01), TP (*p <* 0.05) and TC (*p <* 0.01) (Table 2). Therefore, the addition of root exudates affected not only soil acidity and alkalinity, but also electrical conductivity and soil nutrients (including carbon, nitrogen and phosphorus). Their interaction between the two factors (SRE × AMF) had significant effects on soil pH (*p <* 0.01), AP (*p <* 0.001) and TC (*p <* 0.05) (Table 2). 

### 3.5. The Correlation Between the Characteristics of L. chinensis and the Soil

The results of the multivariable stepwise regression analysis showed that ramet number of *L. chinensis* positively correlated with soil AP and with EC, but negatively related to soil pH (Table 3). There was a positive correlation between aboveground biomass of *L. chinensis* with soil AP. Underground biomass was negatively related to spore density (SD) of AMF. Shoot and root TN showed significant correlations with both soil AN and mycorrhizal infection rate (IR).

## 4. Discussion

### 4.1. Effects of Root Exudates on Soil Properties and AMF

It is suggested that the magnitude of allelopathic substance on soil properties obviously highly depended on their concentration [53]. We had found that the higher concentrations of root exudates significantly reduced soil available nitrogen (AN), available phosphorous (AP), total nitrogen (TN) (Figure 3c–e). These results were similar to the findings of the previous studies, which found that the contents of soil total nitrogen and phosphorus decreased significantly in rhizosphere soil of *S. chamaejasme* [41,54]. Besides, the improvement of low-concentration root exudates on soil AP was consistent with the results of Cesco et al. (2012) [53]. The flavonoids secreted by the roots of *S. chamaejasme* could indirectly increase the soil AP through affecting the colonization of the AMF [21]. Shen et al. (2002) reported that in common bean organic acids might be secreted and could mobilize P from Al- and Fe-bound phosphates, and hence, increase soil AP [55]. Generally, the mechanism how *S. chamaejasme* root exudates affect soil nutrients was quite complicated, which might closely relate to the composition and concentration of the root exudates, soil microorganisms, enzyme activity, and litter decomposition [53,56,57,58].

Previous study reported that allelopathic substance (i.e., terpenes and flavonoids) of a poisonous plant, *Solidago canadensis* L., could increase soil pH [59]. We also found that high concentration of *S. chamaejasme* root exudates significantly increased soil pH. *S. chamaejasme* roots contain flavonoids, lignans, and terpenes and alkaloids [15,16,17]. *S. chamaejasme* root exudates collected from in vivo culture in our experiments were alkalescent (pH = 9.28) which could partially explain the increase of soil pH. Soil pH can directly or indirectly affect the availability of soil nutrient elements, soil microbial composition, enzyme activity, and the metabolism of allelochemicals [50,60,61,62]. Therefore, the alkalescent solution of root exudates may potentially explain why the root exudates had altered the soil carbon, nitrogen, and phosphorus contents.

It has been reported that plant allelochemicals or root exudates also can affect the composition and structure of microbial communities in the rhizosphere soil [44,63,64], but the conclusions are not coincident. The secondary metabolites of the invasive plant *S. canadensis* might promote its competitiveness by enhancing its own AMF symbionts [65]. Root exudates from *Allium sativum* L. could inhibit the growth of the mycelium and germination of zoospores of *Phytophthora capsici* [66]. Our results showed a particular inhibitory effect of *S. chamaejasme* root exudates on the AMF infection rate and spore density (Table 1, Figure 2). Many secondary metabolites have been isolated from the root of *S. chamaejasme* by organic solvent extraction such as coumarins, flavonoids, lignans, and diterpenes [15,16]. The bioactivity assay showed that some of these daphneolone analogues synthesized by *S. chamaejasme* were potentially active against plant pathogenic fungi, such as *Rhizoctonia solani*, *Gibberella zeae*, *Bipolaris maydis*, *Sclerotia sclerotium*, and *Botrytis cirerea* [67]. Therefore, we speculated that the components of *S. chamaejasme* root exudates might inhibit AMF infection. No AMF infection rate in the root system of *S. chamaejasme* in the field also supported such a speculation [40].

### 4.2. Effects of Root Exudates on L. chinensis

*S. chamaejasme* root exudates could affect the growth and development of the surrounding plants through allelopathy. The lower concentration had shown a tendency of positive effects on *L. chinensis* growth, whereas the higher concentrations had negative effects (Figure 1; Table 1). These results reinforced the previous findings of the effect of *S. chamaejasme* on the seedling growth of adjacent plants [13]. In our study, the influence of root exudates on the growth of *L. chinensis* can be better explained by the changes in rhizosphere soil nutrients. Low-concentration promotion and high-concentration inhibition were the general rules of the effects of root exudates on the growth of *L. chinensis* (Figure 1) and nutrient availability (Figure 2). Soil nutrient availability, especially nitrogen and phosphorus, is often a limiting factor for plant growth in grassland ecosystems [68,69], and is closely related to the nutrient content of the plant [70]. Our stepwise regression analysis also further proved that AN and AP were one of the critical factors affecting the ramet numbers and the above-ground biomass of *L. chinensis* (Table 3). Therefore, in consistent with our hypothesis, *S. chamaejasme* root exudates appeared to affect the growth and nitrogen accumulation of *L. chinensis* mainly by changing the soil pH and soil nutrient availability. In summary, this supported our first hypothesis.

### 4.3. Inoculation Effects of AMF

The mycorrhizal infection rate is an important parameter to evaluate the symbiotic relationship between AMF and host plants [71]. The successful colonization of AMF in many clonal plants, such as *Prunella vulgaris* L. and *Potentilla reptans* L. has been reported [72,73,74]. The high AMF colonization rate in *L. chinensis* (87.91~100%) indicated that AMF could establish a strong symbiotic relationship with the root system of *L. chinensis*. AM symbiosis could increase photosynthetic and water use efficiency, and further improve host plant growth [75]. A case study showed AMF inoculation promoted the formation of new ramets in *Hedernepalensis* var. *sinensis*. However, AMF failed to affect the biomass of some stoloniferous clonal species, such as *Trifolium repens* L. and *Gnetum montanum* Markgr. [47]. Our results also did not find a positive effect of AMF inoculation on the growth of *L. chinensis* (Table 1). These inconsistent conclusions determined that AMF inoculation effects on plant ecological performance might be species-specific. The relatively slow process of AMF from inoculation to the colonization in a host plant might lead to a time-lag effect of AMF in promoting plant growth.

Although no significant effects on population performance (i.e., ramet number and biomass) of *L. chinensis,* AMF inoculation had a significant effect on physiological indexes, i.e., shoot nitrogen content (Figure 1; Table 1). A number of studies had also shown that AMF could increase plant absorption of nitrogen [27,28,29,30]. After exogenous application of 15N, the nitrogen content in the shoot and root of mycorrhizal plants was higher than that of the control [76]. AMF inoculation could improve the formation of a large number of extracellular mycelia to increase the root area of plants. Meanwhile, because of ammonium transporters of AMF, extracellular mycelium could facilitate the absorption of NH_4_^+^ and NO_3_^−^ from the soil, which was transferred to intracellular hyphae hydrolysis into the plants [77,78].

In addition, AMF inoculation significantly affected the TN, AP, TP, and TC (Table 2) in this study. AMF are usually known as obligate biotrophs that cannot utilize organic phosphorus, but they can release C-rich compounds into the soil, thereby promoting phosphorus-solubilizing bacteria to mineralize phosphorus [79]. Additionally, AMF might increase soil AP by improving the soil bacterial diversity and phosphorus-solubilizing bacteria (*Acidobacteria*) abundance [80]. Therefore, we suggested that AMF may indirectly affect plant growth by regulating soil chemical characteristics, especially the availability of phosphorus. According to the increasing effects on the mycorrhizal infection rate, spore density, and the shoot nitrogen content, our results suggested that AMF may have potentially facilitate the growth of *L. chinensis* by increasing nitrogen uptake and utilization rate of its root system.

### 4.4. Interaction Effects

The interaction between of *S. chamaejasme* root exudates and AMF significantly changed pH, AP, and TC of rhizosphere soil (Table 2). AMF altered the effects of the additions of the *S. chamaejasme* root exudates on soil properties (Table 1). These results verified the second hypothesis of our study. Soil microbes play an essential role in altering the activities of allelochemicals and can degrade some allelopathic active substance into non-allelopathic active [81]. Studies have shown that the connectivity of mycorrhizal fungi hyphae may play a key role in regulating the movement of allelochemicals in soil and affecting their bioactive zones [82]. The mycelium of AMF could mediate allelochemical transport, such as juglone [83].

Furthermore, the change of soil pH is closely related to the availability and supply capacity of soil mineral elements. Therefore, the inoculation of the AMF might have changed the effects of the *S. chamaejasme* allelochemicals through some direct or indirect approaches. Additionally, AMF infection rate (IR) and rhizosphere spore density (SD) were important factors in affecting the underground biomass and nitrogen content of the plant (Table 3). Hence, these results provided reliable evidence for the regulatory ability of AMF in the growth of *L. chinensis*. Therefore, in consistent with our third hypothesis, our results suggested that AMF have the potential to regulate the allelopathic effects of *S. chamaejasme* and the relationship between *S. chamaejasme* and *L. chinensis*.

Root exudates of poisonous plants may comprise a diverse of chemical compounds, which can differentially affect growth of neighboring plants [84]. However, there was no report about the chemical compounds of *S. chamaejasme* root exudates that were collected in vivo. In previous studies, many secondary metabolites have been isolated from the roots and rhizosphere soil of *S. chamaejasme* by organic solvent extraction, including coumarins, flavonoids, lignans, and diterpenes [15,16,17]. We speculated that the main active components of root exudates in this study might be the same as the results that have been identified above. As we know releasing root exudates is the primary way in which poisonous plants interact with neighboring plants in natural grasslands. In our study, the root exudates instead of the organic solvent extracts of *S. chamaejasme* could better simulate a natural process that exists in the grassland ecosystem. The chemical composition of *S. chamaejasme* root exudates and their differential effects still need further research in the future.

## 5. Conclusions

Our study highlighted the regulatory roles of AMF in mediating the interspecific relationship between poisonous and neighboring plants. In this study, we investigated the influence of *S. chamaejasme* root exudates, AMF inoculation, their interaction on the growth of *L. chinensis* and soil physicochemical properties in the rhizosphere. Firstly, we concluded that the *S. chamaejasme* root exudates had allelopathic effects on the growth of *L. chinensis*, and the effects were closely related to the concentration of the root exudates. Low concentration of *S. chamaejasme* root exudates positively improved the growth of *L. chinensis*, whereas high concentrations showed the negatively inhibiting effects. High concentration of root exudates also significantly reduced soil nutrients. The critical factors in affecting the growth of *L. chinensis*. Secondly, AMF could mediate the allelopathic effects of *S. chamaejasme* via changing nutrient availability. These results indicated that AMF could interact with the root exudates to change some soil chemical characteristics and then potentially regulate the interspecific relationship between poisonous plants and neighboring forage grasses. Taken together, our study provided empirical evidence revealing the allelopathic mechanism of *S. chamaejasme* and the regulatory effects of AMF on the interspecific relationship between poisonous plants and neighboring plants. The combination of the allelopathy of poisonous plants and the regulation of AMF will be meaningful to recover the dominance of palatable grasses in persistent degraded grassland ecosystems.

## Figures and Tables

**Table 1:** The ANOVA of the addition of *S. chamaejasme* root exudates (SRE) and inoculation with and without AM fungi (AMF) on the growth characteristics (ramet number, RN; rhizome length, RL; aboveground biomass, AB; underground biomass, UB) and nitrogen (N) contents of *L.chinensis*, infection rates and spore densities of AMF.

Treatment		*L. chinensis* Characteristics						AMF Characteristics	
	df	RN	RL	AB	UB	Shoot N	Root N	Infection Rate	Spore Density
SRE	3	10.103 ***	0.367	2.913 *	0.236	3.897 *	5.123 **	4.399 **	2.777 *
AMF	1	2.888	0.507	0.371	3.047	7.995 **	5.027 *	847.979 ***	279.477 ***
SRE×AMF	3	1.014	0.956	0.263	0.103	0.876	0.423	0.714	2.047

* *p* < 0.05, ** *p* < 0.01, *** *p* < 0.001.

**Table 2:** The ANOVA of the addition of *S. chamaejasme* root exudates (SRE) and inoculation with and without AM fungi (AMF) on soil characteristics (electrical conductivity, EC; available phosphorous, AP; available nitrogen, AN; total nitrogen, TN; total phosphorous, TP; total carbon, TC).

Treatment		Soil Chemical Characteristics						
	df	pH	EC	AN	TN	AP	TP	TC
SRE	3	44.127 ***	71.023 ***	66.712 ***	34.180 ***	34.650 ***	1.571	38.680 ***
AMF	1	2.744	2.751	2.066	4.749 *	9.516 **	4.381 *	5.592 **
SRE × AMF	3	4.948 **	1.164	0.215	0.445	6.710 **	0.435	3.309 *

* *p* < 0.05, ** *p* < 0.01, *** *p* < 0.001.

**Table 3:** Multivariable stepwise regression analysis of the main soil and AMF parameters influencing the characteristics of *L. chinensis.* EC: electrical conductivity, AP: available phosphorous, AN: available nitrogen, IR: infection rate.SD: spore density.

	Results	R^2^	*p*
Ramet number	1.44 AP −7.937 pH −0.17 AN +0.071 EC +70.52	0.412	0.000
Aboveground biomass	0.15 AP +2.319	0.065	0.041
Underground biomass	−0.004 SD +2.647	0.080	0.023
Shoot nitrogen content	0.032 IR +0.022 AN +28.580	0.239	0.000
Root nitrogen content	0.022 IR +0.034 AN +18.084	0.357	0.000
